# Performing in spite of starvation: How Saccharomyces cerevisiae maintains robust growth when facing famine zones in industrial bioreactors

**DOI:** 10.1111/1751-7915.14188

**Published:** 2022-12-08

**Authors:** Steven Minden, Maria Aniolek, Henk Noorman, Ralf Takors

**Affiliations:** ^1^ Institute of Biochemical Engineering University of Stuttgart Stuttgart Germany; ^2^ Royal DSM Delft The Netherlands; ^3^ Department of Biotechnology Delft University of Technology Delft The Netherlands

## Abstract

In fed‐batch operated industrial bioreactors, glucose‐limited feeding is commonly applied for optimal control of cell growth and product formation. Still, microbial cells such as yeasts and bacteria are frequently exposed to glucose starvation conditions in poorly mixed zones or far away from the feedstock inlet point. Despite its commonness, studies mimicking related stimuli are still underrepresented in scale‐up/scale‐down considerations. This may surprise as the transition from glucose limitation to starvation has the potential to provoke regulatory responses with negative consequences for production performance. In order to shed more light, we performed gene‐expression analysis of Saccharomyces cerevisiae grown in intermittently fed chemostat cultures to study the effect of limitation‐starvation transitions. The resulting glucose concentration gradient was representative for the commercial scale and compelled cells to tolerate about 76 s with sub‐optimal substrate supply. Special attention was paid to the adaptation status of the population by discriminating between first time and repeated entry into the starvation regime. Unprepared cells reacted with a transiently reduced growth rate governed by the general stress response. Yeasts adapted to the dynamic environment by increasing internal growth capacities at the cost of rising maintenance demands by 2.7%. Evidence was found that multiple protein kinase A (PKA) and Snf1‐mediated regulatory circuits were initiated and ramped down still keeping the cells in an adapted trade‐off between growth optimization and down‐regulation of stress response. From this finding, primary engineering guidelines are deduced to optimize both the production host's genetic background and the design of scale‐down experiments.

## Abstract 2

An unprepared exposure to famine zones in industrial carbon‐limited processes can trigger the environmental stress response in yeasts. Repeated exposure will lead to the emergence of a new phenotype optimized to grow more efficiently via increasing ribosome biogenesis and decreasing stress response mechanisms. The insights are based on a transcriptomic study of stimulus‐response experiments in glucose‐limited chemostats.

## INTRODUCTION

Saccharomyces cerevisiae is a time‐tested and widely applied host in the biotech industry. Its central status as a cell‐factory is rooted in an extensive knowledge base, advanced and facilitated genetic engineering, unproblematic valorization of biomass as a byproduct and foremost, robustness to diverse industrial conditions (Nielsen, 2019). The latter is based on the yeasts' ability to adapt to a wide array of ecological niches (Goddard & Greig, 2015; López‐Maury et al., 2008), which is both a blessing and a curse for bioprocesses development. While ample adaptation mechanisms made the yeast a preferred platform organism for many bioprocesses, its flexibility comes at a price. Bioprocesses are typically developed in a homogeneous environment in lab‐scale studies. In contrast, the industrial habitat is characterized by imperfect mixing since maintaining equal mean broth circulation time with increasing tank volume poses an infeasible endeavour (Junker, 2004; Uhl & Von Essen, 1986). Resultant dynamic gradients, *for example*, of primary nutrients, constantly challenge the adaptive capacity of the cells even leading to non‐expected regulation phenomena that may cause the deterioration of expected TRY (titre, rate, yield) criteria (Crater & Lievense, 2018; Enfors et al., 2001; Takors, 2016). This mirrors the interaction of multi‐level regulation programs covering allosteric enzymatic control, transcriptional, translational and post‐translational responses finally leading to physiological changes. Notably, each regulatory level possesses inherent response and relaxation times which overlap finally creating the integral response on external stimuli (Delvigne & Goffin, 2014; Wehrs et al., 2019). Hence, scale‐up effects are the outcome of the complex interactions between production‐scale hydrodynamic heterogeneities and multi‐level yeast responses.

Carbon‐limited fed‐batch strategies are widely adopted to ensure efficient conversion of substrate to product, for instance, in a baker's yeast production. Feed rates are designed to allow fast growth while avoiding resource spillage through overflow metabolism. As a consequence, consumption times for highly diluted substrates may be shorter than the convective supply of said substrates leading to substrate depletion in poorly mixed zones of the bioreactor or far away from the inlet point (Lara et al., 2006). Inherently, substrate gradients (e.g. for glucose) creating excess and scarcity are likely to occur as confirmed experimentally and by simulation investigating the industrial bioreactor (George et al., 1998; Haringa et al., 2017; Sarkizi Shams Hajian et al., 2020). *Saccharomyces cerevisiae* senses variable substrate supplies via a plethora of multilayered and interconnected signalling cascades. Extracellular glucose levels are detected via the Gpr1/Ras2‐cAMP‐dependent protein kinase A (PKA) and Rgt2/Snf3‐protein kinase B (PKB) nutrient kinases (Busti et al., 2010; Kim, Roy, et al., 2013). The sensing of intracellular glucose pools is directly mirrored by hexokinase activity and indirectly by the adenylate energy charge, AEC, through the Snf1/AMP‐activated protein kinase (AMPK) network (Coccetti et al., 2018). The status of low ATP availability, that is, low AEC, is transduced via Snf1 to the rapamycin kinase complex I (TORC1) which regulates the growth rate together with PKA (Kunkel et al., 2019; Wullschleger et al., 2006). Further downstream, these regulatory nodes orchestrate the phosphorylation status of central transcription factors (TFs) finally translating external stimuli into well‐adjusted microbial responses (Petrenko et al., 2013; Plank, 2022).

What determines the biological output from the above regulatory network is the combination of amplitude, frequency and dwell time with respect to the exposure to a certain glucose concentration. Responses may be subtle, short‐termed but well‐buffered energetic imbalances or even fatal growth arrests (Bisschops et al., 2017; Verma et al., 2013). In any case, they are likely to deteriorate the productivity of engineered cells to produce the targeted product. Knowledge‐driven downscaling aims to mimic related scenarios already in lab‐scale for identifying proper prevention strategies (Delvigne & Noorman, 2017; Straathof et al., 2019; Takors, 2016). As a prerequisite of modern approaches, production‐scale information is deduced from computational fluid dynamic (CFD) studies (Haringa et al., 2016; Lapin et al., 2004). Adding the biological phase to the flow field via cellular reaction dynamics (CRD) models, which are derived from stimulus–response experiments (SRE), enables the in silico characterization of relevant environmental stimuli (Penia Kresnowati et al., 2005; Zieringer & Takors, 2018). Finally, coupled CFD‐CRD simulation results govern the quantitative design of both, realistic scale‐down reactors and strains with increased process robustness (Haringa et al., 2017; Kuschel & Takors, 2020; Wang et al., 2020).

More and more studies highlight the prevalence of starvation zones in bioreactors that occurred distant from the feed zone in fed‐batch processes (Haringa et al., 2016; Ho et al., 2022; Kuschel & Takors, 2020; Nadal‐Rey et al., 2021). Remarkably, SRE‐data covering the transition between carbon limitation and starvation are scarce, whereas the opposite, that is, sudden shifts towards glucose excess, were extensively studied in the past (Kresnowati et al., 2006; Suarez‐Mendez et al., 2017; Theobald et al., 1997; Verhagen et al., 2022; Wu et al., 2006). The latter may reflect the fundamental interest in the Crabtree effect and its relevance for multiple metabolic scenarios (de Alteriis et al., 2018). However, such stimuli studies do not mimic the predominant conditions in large‐scale bioreactors. Consequently, we set out to complement the current database with kinetic studies investigating the endometabolome after glucose shifts from limitation to starvation (Minden et al., 2022). In the referenced work, the metabolome of *S. cerevisiae* revealed a short‐term strategy optimized to uphold anabolic needs at the expense of catabolic capacities when entering famine zones. Significantly increased biomass‐specific energy demands after repeated exposure to the same glucose gradient raised the question how the stimulus is propagated in the eukaryotic regulatory network. Using next‐generation‐sequencing data, this study investigates gene‐expression dynamics to answer two questions: (i) How does a yeast population respond to the first‐time occurrence of glucose scarcity and (ii) how is the regulatory landscape shaped after complete adaptation towards the dynamic production environment?

## EXPERIMENTAL PROCEDURES

### Strain maintenance and seed culture conditions

*Saccharomyces cerevisiae* CEN.PK 113‐7D (Nijkamp et al., 2012) was kindly provided by Royal DSM N.V. and preserved as a 30% (v/v) glycerol stock at −70°C and maintained on yeast extract peptone dextrose (YPD) agar plates at +4°C. Seed cultures were prepared by inoculating 5 ml YPD broth with single colonies in a glass vial followed by an 8‐h incubation at +30°C on an orbital shaker operated with 120 rpm. The whole culture was pelleted and transferred to 110 ml of a synthetic medium in a 1000 ml baffled shake flask and incubated under identical conditions overnight. The medium was modified from Verduyn et al. (1992) to support carbon‐limited growth in continuous culture with 22.5 g L^−1^ glucose. In brief, the referenced salt concentrations were increased threefold and the trace element and vitamin stock solutions were increased twofold.

### Bioreactor setup and continuous operation mode

Aerobic, continuous fermentations were carried out in a stainless steel benchtop bioreactor (Bioengineering) with a liquid working volume of 1.7 L. The culture was supplied with sterile ambient air through a fumigation frit positioned at the reactor bottom with a constant flow rate of 0.5 vvm. Broth homogenization and bubble dispersion were ensured with two six‐blade Rushton‐type impellers operated constantly at 800 rpm equalling a gassed, volumetric power input of 7.1 W kg^−1^ to yield a circulation time of 0.1 s (Appendix S1: Tables S1 and S2). The relative dissolved oxygen concentration was determined with an optical pO_2_ probe (PreSens) and never decreased below 70%. Broth temperature was controlled at +30°C with electrical heating and water cooling rods and monitored with a Pt100 probe (Bioengineering). The tank was operated at an absolute pressure of 1.3 bar, which was maintained with a needle valve attached at the off‐gas filter element exit. Two molar potassium hydroxide kept the broth pH at 5.00 using a Mettler Toledo probe. A continuous supply of Struktol J 674 antifoam agent (Schill und Seilacher) with a pump rate of 30 μl h^−1^ was realized with a LA‐120 syringe pump (IDL GmbH) to pre‐emptively avert foaming. Molar oxygen and carbon dioxide fractions in the off‐gas were logged every minute with BCP‐O_2_ and BCP‐CO_2_ sensors (BlueSens). All in‐ and outgoing liquid flows were conveyed with U‐120 peristaltic pumps (Watson‐Marlow). Rapid sampling was enabled using semi‐automated sampling devices based on time‐relay controlled opening of a pinch valve (Minden et al., 2022).

Bioreactors were inoculated with 100 ml seed culture and the continuous phase was initiated after a rapid increase in the pO_2_ signal marked the end of the batch phase. During continuous operation mode, the medium was fed at a fixed rate of 2.83 ml min^−1^ to yield a dilution rate of 0.1 h^−1^ via mass balancing of the whole fermenter through the harvest pump. The feed medium was constantly homogenized with a magnetic stirrer to prevent gradient formation.

### Experimental design

**FIGURE 1:**
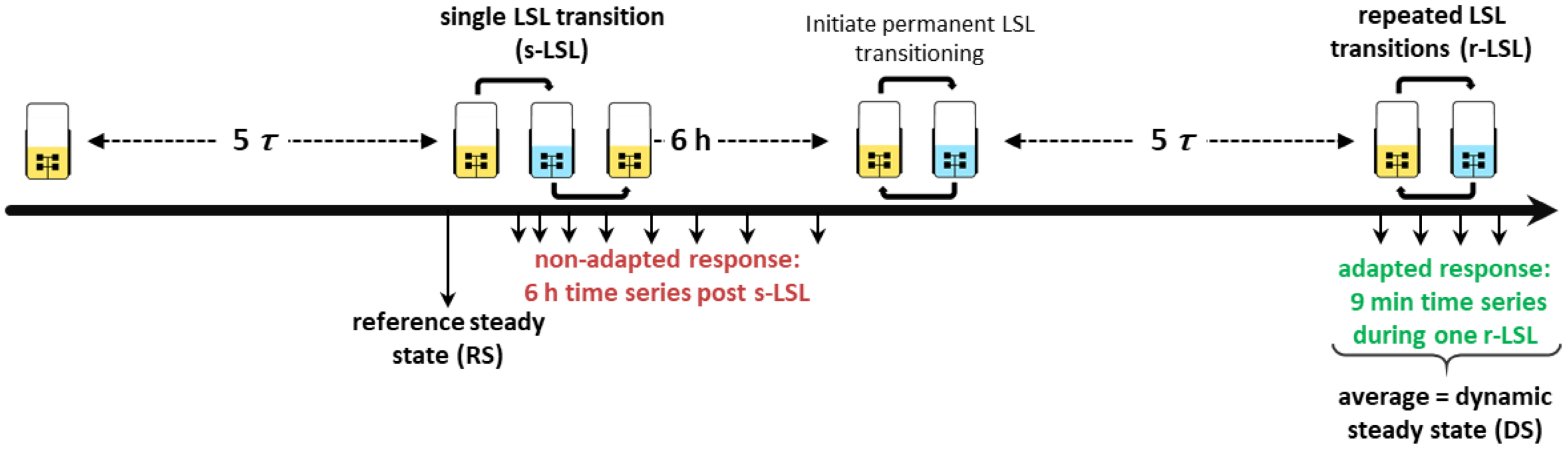
Process design of the chemostat experiment. *τ*, residence time.

Both, non‐adapted and adapted starvation response experiments were conducted in the same chemostat process according to the process design depicted in Figure 1. First, the reference steady state (RS) was sampled after five residence times of constant $Q_{O_{2}}$ and $Q_{{\mathrm{CO}}_{2}}$ conjointly marking time point 0 min of the non‐adapted time series. Subsequently, the feed was interrupted for 2 min causing a transition from limitation to starvation back to limitation (LSL) and the stimulus–response was monitored for up to 6 h (denoted post s‐LSL, s for single). Second, the dynamic steady state (DS) was characterized after five residence times of repeated LSL (r‐LSL) transitioning. During this phase, the feed was operated in 9‐min LSL‐cycles with the feed inactive for 2 min and active for 7 min equalling a 9 min r‐LSL cycle time. The active feed rate was adjusted to 3.64 ml min^−1^ resulting in a net dilution rate of 0.1 h^−1^. Samples for the adapted response were drawn over one representative 9‐min cycle and steady‐state DS was expressed as the average over one cycle.

### Sample follow‐up and analytical procedures

All samples were measured in groups of technical triplicates and values reported in this study are expressed as the arithmetic mean ± standard deviation of technical means from three independent fermentation experiments. Carbon, nitrogen and available electron balances closed within ±3.6% at any sample point (see Appendix S1: Figure S1).

Dry matter of biomass (DMB) was quantified gravimetrically via vacuum‐filtration of 5 ml degassed fermentation broth through desiccated and tared membrane filters (Ø 47 mm, Type 154; Sartorius). The filter cake was washed with 15 ml deionized H_2_O and dried in a heating chamber at +70°C until mass remained constant after occasional weighing.

To assess extracellular glucose, broth was directly withdrawn into an open syringe and squeezed through a PES filter element (Ø 30 mm, 0.22 μm pore size, ROTILABO®; Carl Roth) within less than five seconds. The supernatant was flash‐frozen in liquid nitrogen and stored at −70°C until analysis. Glucose was quantified with a UV‐based enzyme test kit (art. no. 10716251035; r‐biopharm AG) without sample dilution according to the manufacturer's instructions.

Intracellular glycogen determination was following the protocol originally published by Parrou and Francois (1997) and modified by Suarez‐Mendez (2015) for rapid quenching. In brief, 1.5 ml broth was collected in 10 ml of <−40°C methanol and subsequently centrifuged for 5 min at −11°C under 5000 *g*. The resulting pellet was flash‐frozen and stored at −70°C. Upon thawing, pellets were rendered permeable in 0.25 ml 0.25 M sodium carbonate heated to +95°C for 3 h in a water bath. Next, optimal conditions for enzymatic glycogen conversion to glucose were established by adding 0.15 ml M acetic acid and 0.6 ml 0.2 M sodium acetate (pH 5.2, adjusted with acetic acid). 0.48 ml of the resulting suspension was mixed with 20 μl of α‐amyloglucosidase (~70 U ml^−1^, cat. number: 10115; Merck) and incubated for +57°C for at least 12 h. Finally, the resulting suspension containing liberated glucose was separated from cellular debris via centrifugation (2 × 10^4^ *g*, 1.5 min) and quantified as described above.

Intracellular total RNA levels were assessed based on the method described by Sasano et al. (2017). One millilitre of fermentation broth was transferred into a tube containing chilled 0.5 ml 1 M perchloric acid. The sample was immediately homogenized and placed for 20 min in a water bath maintaining +70°C. Subsequently, the sample was mixed with 0.5 ml of 1 M K_2_HPO_4_ and the formed precipitate was removed via centrifugation (2 × 10^4^ *g*, 1.5 min). The supernatant was flash frozen and stored at −70°C until RNA determination with a Nano‐Drop ND‐1000 (NanoDrop Technologies), which was blanked against a solution containing 0.25 M perchloric acid and 0.25 M K_2_HPO_4_.

### Estimation of $q_{\mathrm{ATP}}$

Biomass‐specific ATP formation rate was estimated based on its stoichiometric relationship with oxygen uptake and glucose consumption according to $q_{\mathrm{ATP}}=2\cdot q_{S}+2\cdot \frac{P}{O}\cdot q_{O_{2}}$ with an assumed $\frac{P}{O}$ ratio of 1.08 (Van Den Brink et al., 2008). The specific oxygen uptake rate was calculated after deconvolution of the off‐gas sensor readout due to the volume of tubing and foam traps causing significant detection delays. The deconvolution method from Theobald (1995) was applied and has been described in detail recently (Minden et al., 2022).

### Total RNA extraction

Total RNA extraction was performed using the Quick‐RNA Fungal/Bacterial Miniprep Kit (R2014; Zymo Research) following the manufacturer's instructions with slight modifications. Prior to sampling, ZR BashingBead™ lysis tubes were prepared with 0.4 ml RNA lysis buffer and 0.1 ml DNA/RNA Shield™ agent (Zymo Research; not provided with the kit). During the experiment, 0.25 ml fermentation broth was instantly added to the prepared lysis tube, vigorously shaken by hand and flash‐frozen in liquid nitrogen (all <10 s). This sampling routine yielded maximally 55 mg wet biomass (assuming a dry:wet matter of biomass correlation of 0.21 estimated from Aon et al., 2016) which is within the range of the recommended upper loading limit of 50–100 mg wet weight. Samples were stored at −70°C and extracted in batch from all three fermentations. The extraction protocol was started by thawing the samples fifty‐fifty and subsequently homogenizing the sample in a Precellys 24 tissue homogenizer (Bertin Technologies) for two times 20 s at maximum speed with a 10 s break in between. All subsequent steps were performed according to the manufacturer's instructions. At the end of the protocol, total RNA was eluted with 60 μl DNase/RNase‐free H_2_O and each sample as split in two 30 μl aliquots.

### Library preparation and RNA‐sequencing

One aliquot from each sample was shipped for mRNA sequencing to GENEWIZ. Initial quality checks using the Agilent 2100 BioAnalyzer instrument (Agilent) revealed high integrity of all samples with uniform RIN (RNA integrity number) values ≥9.9. Next, cDNA libraries were synthesized after polyA selection was performed to enrich mRNAs. Libraries were finally sequenced as paired‐end reads of 150 base pair length on a NovaSeq 6000 platform (Illumina) with a sequencing depth of 2 × 10^7^ paired‐end reads per sample.

### Processing of sequencing data

Sequencing results were received in the *.fastqsanger* format and uploaded on a local galaxy server instance (Afgan et al., 2018). First, the sequencing quality was assessed for each file individually using *FastQC* v. 0.72 (Andrews, 2010). Adapter sequences were removed using *Trimmomatic* v. 0.38.0 (Bolger et al., 2014) for paired‐end reads with default settings. The trimmed sequence files were then aligned against the *S. cerevisiae* CEN.PK113‐7D reference genome (GCA 000269885 – ASM 26988 v1) accessed from the ENSEMBL database (Howe et al., 2021) using the *TopHat* v. 2.1.1 (Kim, Pertea, et al., 2013) algorithm for paired‐end reads with default settings yielding an overall alignment rate of 86%–93%. Count tables were computed using *featureCounts* v. 1.6.4 (Liao et al., 2014) together with the strain‐specific annotation file *Saccharomyces_cerevisiae.R64‐1‐1.50.gtf*, also obtained from the ENSEMBL database. The generated count tables were merged into a *data.frame* object in the R environment v. 1.4.1106 (R Core Team, 2021) for downstream analysis.

### Differential gene expression analysis

Differential gene expression analysis was conducted using the *DESeq2* v. 1.32.0 R‐package (Love et al., 2014). After transforming the count table into the homoscedastic log_2_‐scale with *rlog*, PCA analysis revealed a significant proportion of variance introduced into the dataset via multiple library preparations and sequencing runs (Appendix S2: Table S1 and Appendix S1: Section A3). Thus, the variables ‘library run’ and ‘sequencing run’ (as a merged variable called ‘libseq’) were introduced into the experimental design matrix. Time series and steady‐state comparison were analysed with the likelihood ratio test (test = “LRT”) and a model reduced by technically introduced variance (for details, see Appendix S1: Figures S2–S4). Genes were considered as differentially expressed with a |log_2_‐fold change| above 0.322 and a false discovery rate (FDR) (Benjamini & Hochberg, 1995) below 1 × 10^−3^. For further analysis, open reading frame identifiers were converted to ENSEMBL gene names using the libraries *AnnotationDBi* v. 1.51.5 and *org.Sc.sgd.db* v. 3.13.0.

### Multidimensional scaling

Classical metric multidimensional scaling (Gower, 1966) was performed to visualize global dissimilarities in the gene expression profiles of all samples. First, log_2_‐scaled count tables were cleaned from technical variance using the function *removeBatchEffect* from the *limma* v. 3.48.3 package (Ritchie et al., 2015). Subsequently, biological replicates were expressed as arithmetic means and only genes with significant differential expression in at least one condition were considered. The resulting table was converted to a Euclidean distance matrix using the *dist* function, transposed and passed to *cmdscale* (k = 3) for a three‐dimensional representation of the sample distances. The functions *dist* and *cmdscale* were called from the *stats* v. 4.1.0 package.

### Cluster and functional enrichment analysis

Time series gene expression data were clustered into groups of genes with similar patterns of log_2_‐fold changes using the *kmeans* function from the *stats* v. 4.1.0 package. The algorithm was operated with a maximum of 1 × 10^3^ iterations around two centroids for the adapted and six centroids for the non‐adapted time series. For each cluster, gene ontology (GO) enrichment was assessed using the YeastEnrichr web interface (Chen et al., 2013; Kuleshov et al., 2019). YeastEnrichr was queried for the ‘GO_Biological_Process_2018’ library (source: http://geneontology.org/; release 2022‐03‐22) and significant terms (FDR < 0.05) were manually curated to avoid redundancy of GO terms. Up‐ and down‐regulated gene lists from the comparison between steady‐states RS and DS were additionally queried for the ‘WikiPathways_2018’ (source: https://www.wikipathways.org; accessed 2022‐04‐15) and the ‘GO_Cellular_Component_2018’ (source: http://geneontology.org/; release 2022‐03‐22). Non‐curated enrichment results can be accessed in the Appendix S2, Tables S4–S9, S11, S12 and S14–S19.

Gene set enrichment analysis (GSEA) was performed with the R package *GAGE* v. 2.42.0 (Luo et al., 2009) to investigate significant differential expression of pre‐defined gene lists. As described previously, log_2_‐scaled count tables corrected for technical variance were used as an input for the *gage* function, which was configured to perform paired comparisons (compare = “paired”). Two‐sample *t*‐test values were used as a proxy for the intensity of gene‐expression changes of the underlying gene set and converted to heat maps using the *ggplot2* package (version: 3.3.6.9000). Literature gene sets were extracted from various sources and transcription factor target lists were obtained from the Yeastract database (Monteiro et al., 2020). All 183 transcription factors available from Yeastract were queried for genes with documented ‘DNA binding and expression evidence’ and converted to a *.gmt* file as an input for the *gage* function. Only literature gene sets and transcription factor target sets that were enriched significantly (FDR <0.05) in at least one condition per GSEA analysis were reported. All input and output tables used in this analysis are accessible in the Appendix S2 (Tables S20–S23; *.gmt* tables were reduced to gene sets which are shown in Figure 7).

## RESULTS

### Characterization of the famine stimulus

**FIGURE 2:**
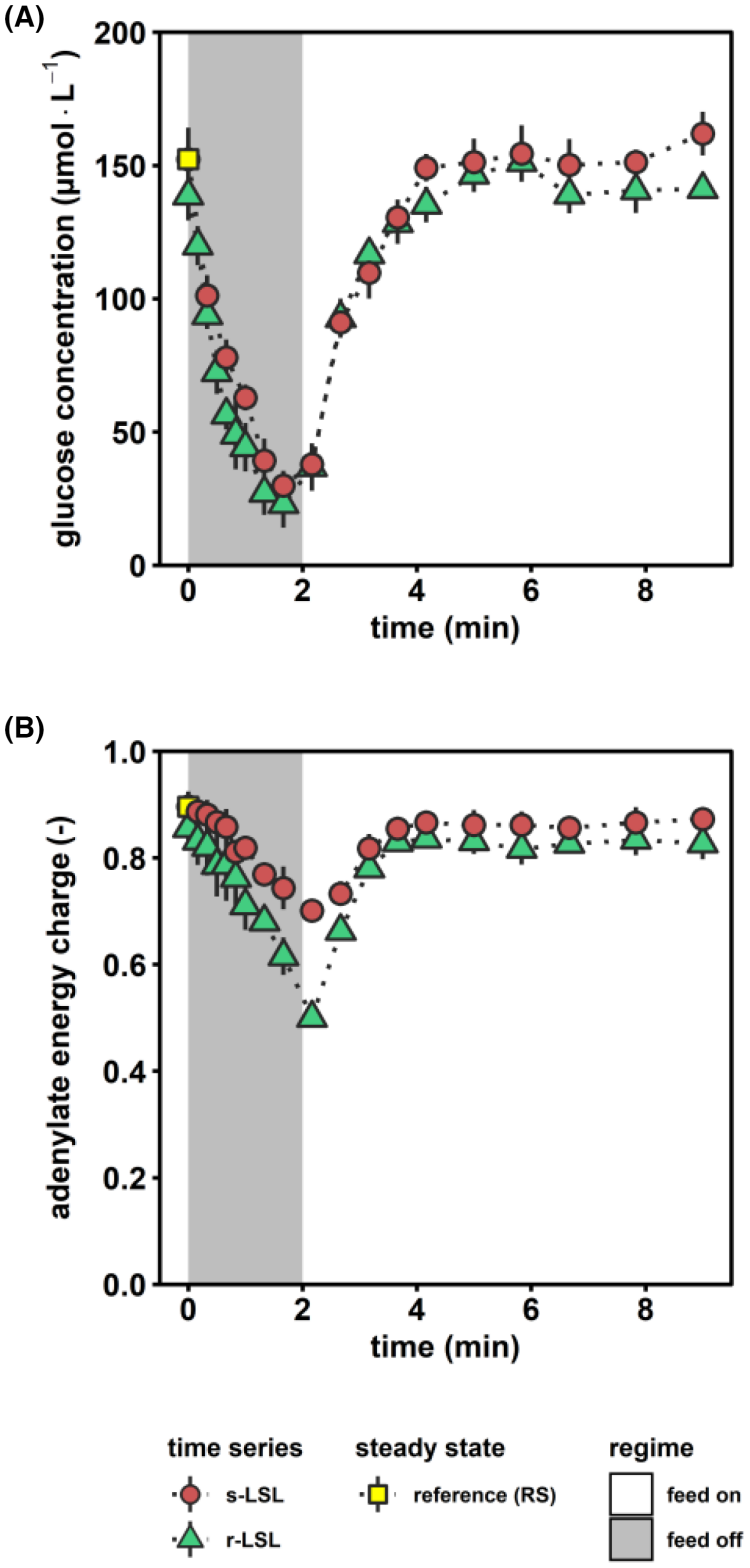
Characterization of the famine stimulus. (A) Extracellular glucose concentration and (B) intracellular adenylate energy charge (AEC) during the course of one perturbation cycle. AEC was calculated based on the methodology reported by Ball and Atkinson (1975). Red circles indicate dynamics following a single (s) LSL‐transition and green triangles depict one representative repeated (r) LSL‐cycle during steady‐state DS. Time point 0 min of s‐LSL is the equivalent of steady‐state RS (yellow squares). All values indicate means ± standard deviation of three biological replicates. The underlying data were previously published in Minden et al. (2022).

Sudden glucose shortages mimicking industrial‐scale famine zones were established by periodic stops of the medium feed during carbon‐limited growth. The 2‐min lasting substrate starvation‐induced glucose reduction from 150 to 30 μmol L^−1^ (Figure 2A). Afterwards, the glucose‐limiting feed scenario was re‐installed finally creating a limitation‐starvation‐limitation (LSL) cycle. Interestingly, resulting glucose profiles were similar for non‐adapted and adapted cells. The latter resulted from the repeated exposure to said LSL cycles (r‐LSL, see Experimental Procedures). During one LSL‐trajectory, biomass‐specific glucose uptake rates ($q_{s}$) were severely curtailed, not exceeding 5% of maximum capacities (9.3 mmol g_DMB_^−1^ h^−1^, from Diderich et al., 1999) for 14% of cycle duration. Given that large‐scale CFD simulations assumed CEN.PK 113‐7D to spend 40% of the time in sub‐5% $q_{s,\max}$ regimes (Haringa et al., 2017), the current experimental approach is qualified as rather mild but still realistic to mimic industrial‐scale glucose depletion scenarios. The calculated adenylate energy charge (AEC) (previously reported in Minden et al., 2022) was monitored as a possible actuator for initiating regulatory energy sensing cascades (Figure 2B). By trend, AEC mirrors the extracellular glucose availability during starvation. The restoration of pre‐stimulus values even occurred slightly faster than the recovery of extracellular glucose levels. Non‐adapted cells decreased their AEC by 0.20 ± 0.03 while amplitudes for adapted cells were almost doubled reaching a minimal value of 0.50 ± 0.01. For a short period, both populations fell below the commonly accepted physiological AEC range of 0.7–0.9 (De La Fuente et al., 2014). This is a rather remarkable observation given that long‐term glucose‐starved yeasts can sustain their adenylate energy charge within the physiological range for up to several hours during the stationary phase (Ball & Atkinson, 1975; Weibel et al., 1974).

### Short‐term starvation evokes macroscopic rearrangements

**FIGURE 3:**
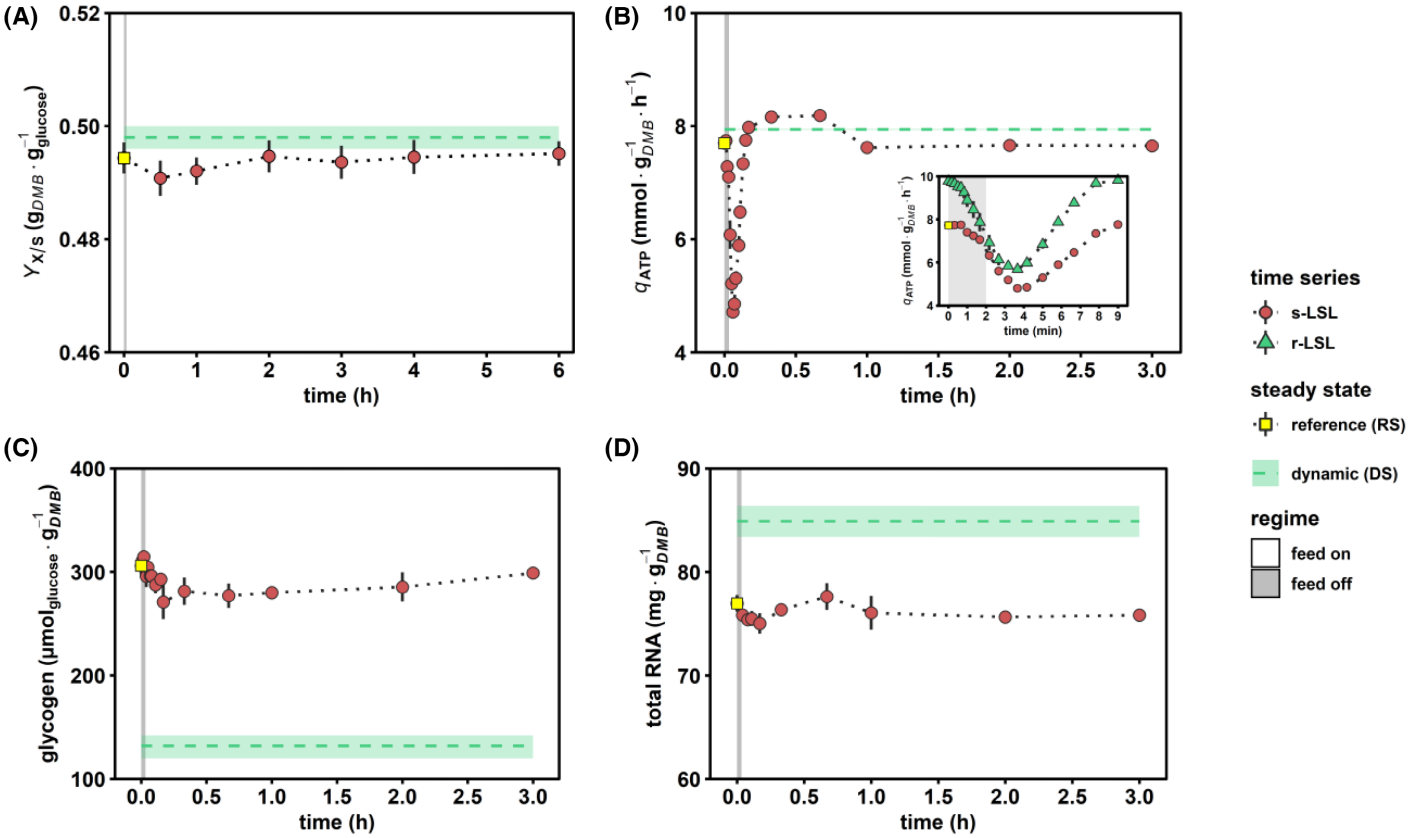
Macroscopic stimulus–response characterization. (A) Biomass‐substrate yield. (B) ATP production rate estimated from oxygen and glucose consumption rates assuming a *P*/*O* ratio of 1.08 (Van Den Brink et al., 2008). The insert plot depicts the short‐term dynamics during one representative LSL‐cycle. (C) Intracellular glycogen and (D) total RNA pool dynamics. Red circles indicate dynamics during and up to 6 h post single (s) LSL and time point 0 min is the equivalent of steady‐state RS (yellow squares). Green triangles depict one representative repeated (r) LSL cycle during steady‐state DS. Steady‐state DS is expressed as the average of dynamic data from r‐LSL cycles (green dashed line) ± standard deviation (light area). All time series values indicate means ± standard deviation of three biological replicates.

Figure 3 compares post‐stimulus data of the unperturbed reference (RS), the steady state after repeated perturbation (DS) and time‐series of non‐adapted cells. Furthermore, the small plot inside Figure 3B depicts two time series that reflect cellular responses during an LSL cycle. This diagram is provided for illustrating that the ‘steady‐state’ after repeated perturbation ‘DS’ rather represents an average of dynamics than a true steady‐state defined by constant state variables.

Notably, the biomass‐substrate yield ($Y_{X/S}$) of RS persisted after long‐term adaptation to alternating glucose availability as indicated by the similar DS (Figure 3A). In part, this was the result of substantial metabolic re‐arrangements in adapted versus RS‐cells, including a reduction of the glycogen pool by 49% and increasing internal RNA abundance from 77.0 ± 1.4 mg g_DMB_^−1^ to 84.9 ± 1.6 mg g_DMB_^−1^ (Figure 3C,D). We quantified total ribonucleic acid as a proxy of ribosomal content, considering that 80% of total RNA in yeast contributes to the assembly of ribosomes as rRNA (Warner, 1999). Thereof, we hypothesized that the 3% rise of $q_{\mathrm{ATP}}$ (*p* < 0.05, Figure 2B) in DS versus RS was necessary to sustain increased translational capacities, which was partially counterbalanced by decreased energy spillage through glycogen‐associated futile cycling.

A similar relation was found during the mid‐term response of unstressed yeast cells post s‐LSL. Within the first 10 min, glycogen pools slightly reduced by 13% to a minimum of 271 ± 29 μmol_glucose_ g_DMB_^−1^, followed by a relatively prolonged repletion phase of 3 h. In parallel, the population showed 5% increased $q_{\mathrm{ATP}}$ 20–60 min post‐stimulus before energy demands relaxed to pre‐stimulus levels. Again, RNA ramp‐up dynamics seemed tightly linked with the temporally increased ATP demands. Following the peak of this non‐adapted response, we found a significant reduction of $Y_{X/S}$ at the 1‐h mark (*p* < 0.05) which eventually recovered. Thus, the temporal observation in this phase might reflect the early initiation and retraction of the phenotypic shift, which is completed after long‐term adaptation in DS.

Interestingly, the immediate intra‐r‐LSL $q_{\mathrm{ATP}}$ response during the representative cycle in Figure 3B revealed a reduction to 4.2 mmol g_DMB_^−1^ h^−1^ which represents a 44% larger amplitude than the non‐adapted population (Figure 3B, insert plot). This observation is consistent with the equally larger AEC amplitudes within one r‐LSL‐cycle (Figure 2B) and points to a larger ATP drain accounting for the intensified translational capacities in adapted cells.

**FIGURE 4:**
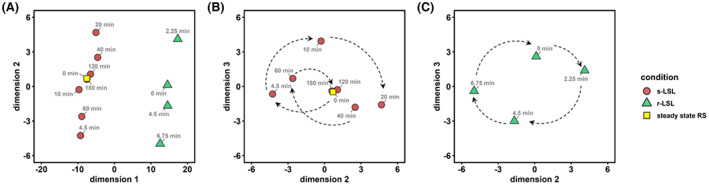
Dissimilarities of significant gene expression patterns in the multidimensional scaling (MDS) space represented by three dimensions. (A) Whole dataset represented by the first two dimensions. (B) MDS plot of the post single (s) LSL time series (red circles + yellow square) based on dimensions 2 and 3. Dashed arrows provide a visual aid to follow the time series (C) Analogous MDS plot of the 9 min repeated (r) LSL time series (green triangles).

Next, we set out to elucidate regulatory phenomena on the gene expression level that govern the observed phenotypic shifts. Figure 4 displays the global analysis of Euclidean distances between all investigated samples using classical metric multidimensional scaling over three dimensions. The analysis of the first dimension distinguishes the grouping of adapted and non‐adapted cells after their exposure to LSL cycles (Figure 4A). The apparent difference in the second dimension is further elucidated if the transcriptional time‐series co‐consider the third dimension (Figure 4B,C). By trend, the s‐LSL exposure pushed the cells quickly away from their steady state within the first 4.5 min and it took about 180 min to return on a spiralled course. This pattern entails oscillating transcriptional dynamics, which reinforce until 20 min before complete relaxation after 180 min. In contrast, we observed a rather circular trajectory for adapted cells. The latter anticipates that a fraction of adapted cells always remained transcriptionally stimulated during the entire course of the experiments.

### S. cerevisiae overloads the strategic response upon first‐time glucose deprivation

**FIGURE 5:**
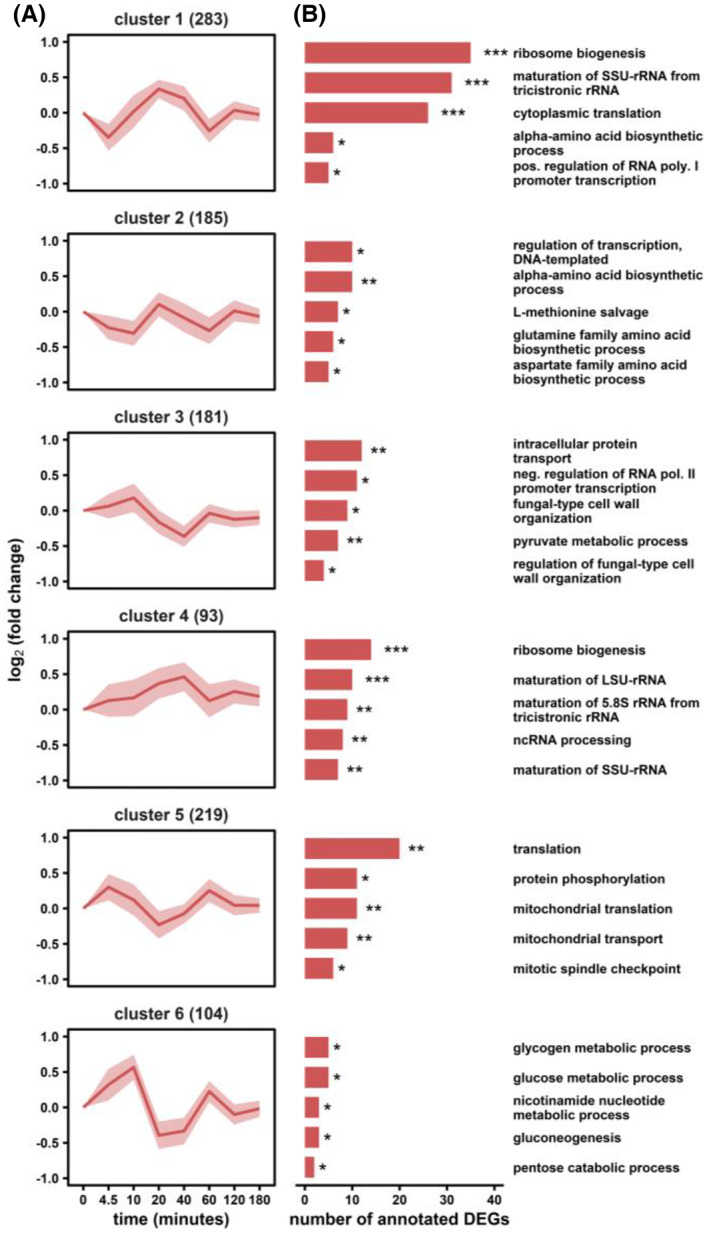
Differential gene expression analysis of the non‐adapted s‐LSL response. (A) Six clusters with similar gene‐expression dynamics are shown with the number of dedicated genes in brackets. (B) Corresponding gene ontology (GO) enrichment analysis. The false discovery rate (FDR) interval is indicated by asterisks for each GO term (* 1 × 10^−5^ ≤ FDR < 5 × 10^−2^; ** 1 × 10^−10^ ≤ FDR <1 × 10^−5^; *** FDR <1 × 10^−10^).

Differential gene expression analysis uncovered 1065 genes accounting for 16% of the reference genome all fulfilling the statistical significance (*p* < 1 × 10^−3^) of differential expression during the 3‐h lasting response upon the s‐LSL stimulus. We grouped the differentially expressed genes (DEGs) into six clusters each featuring similar log_2_fold changes. Furthermore, we assigned co‐regulated genes via the enriched gene ontology (GO) terms (Figure 5). In sum, the clusters confirm the dynamics anticipated from the MDS analysis, which comprises an early transcriptional response followed by an amplified mid‐term amplitude before slowdown.

Three of six clusters were disproportionately enriched with GO terms related to the translation machinery containing one‐third of all 135 ribosomal proteins (RPs) in yeast (Gaikwad et al., 2021). Cluster 4 increased steadily over the first 40 min. Meanwhile, clusters 1 and 5 highlighted other dynamics that are laterally inversed. Whereas cluster 5 showed the early amplification of gene transcripts as described above, cluster 1 disclosed an opposite trend. The two clusters are particularly interesting as a trade‐off between cytoplasmic and mitochondrial translation becomes evident. Several studies outlined that the coordinated redistribution of the costly translation machinery is a crucial feature for building up necessary respiratory capacity under stressful conditions (Bonawitz et al., 2007; Couvillion et al., 2016; Suhm et al., 2018). Further evidence of compartment‐specific resource adjustments is provided by the enriched ‘mitochondrial transport’ ontology in cluster 5. However, we did not identify corresponding up‐regulation of the respiratory chain complex despite our observation of increased ATP dissipation during the observed ramp‐up of RNA content and $Y_{X/S}$. In addition, cluster 1 was enriched with transcriptional inducers of rRNA synthesis from polymerase I anticipating a bilateral relationship between regulatory circuits and their provoked strategic responses.

Co‐regulated amino acid synthesis genes in cluster 2 followed the trajectory of cluster 1 but with a delayed onset and less pronounced fold changes. Both clusters were significantly enriched for ‘alpha‐amino‐acid biosynthesis’ activity, reaching a GO‐term coverage of 46%. For some of the comprised genes, for example, those involved in leucine (*LEU2*, *LEU4*, *LEU9*) and aromatic amino acid biosynthesis (*ARO8*, *ARO7*, *TRP2*, *TRP3*, *TRP5*), the intracellular concentrations of their biosynthetic products qualitatively followed the observed cluster dynamics (Appendix S1: Figure S5). On the other hand, absolute glycogen levels appeared detached from the induction‐repression dynamic of cluster 6 that comprised the related ontology. Nonetheless, this group contained both genes involved in glycogen mobilization (*GPH1* and *GDB1*) and accumulation (*GLC3* and *GDB1*) which may be taken as a hint towards the dynamic activity of futile cycling (Blomberg, 2000; François & Parrou, 2001). We observed a general tendency for the initial repression of genes involved in primary anabolism, while catabolic enzymes from glucose, pentose, and pyruvate metabolic processes followed the opposite trend.

Genes that were annotated to cluster 3 signalled slight activity of stress‐responsive mechanisms. For instance, members of the ‘intracellular protein transport’ comprise chaperone activity such as *SSA1* and *CUR1* or were involved in protein recycling, *for example*, through *VPS29* and *EAR1*. The early induction of transcriptional repressors (‘negative regulation of RNA polymerase II promotor transcription’) may indicate broader macromolecular savings. Furthermore, the LSL‐stimulus triggered changes in cell wall organization and even associated transcription factors (TFs). Taken together, a sudden shift from glucose limitation to starvation prompted *S. cerevisiae* to enter a defensive state preparing for times of scarcity. As this preparatory measure turned out to be premature, a pronounced backlash caused dampened transcriptional bursts up to 2 h post‐stimulus.

### Repeated famine exposure shapes a specialist growth phenotype

**FIGURE 6:**
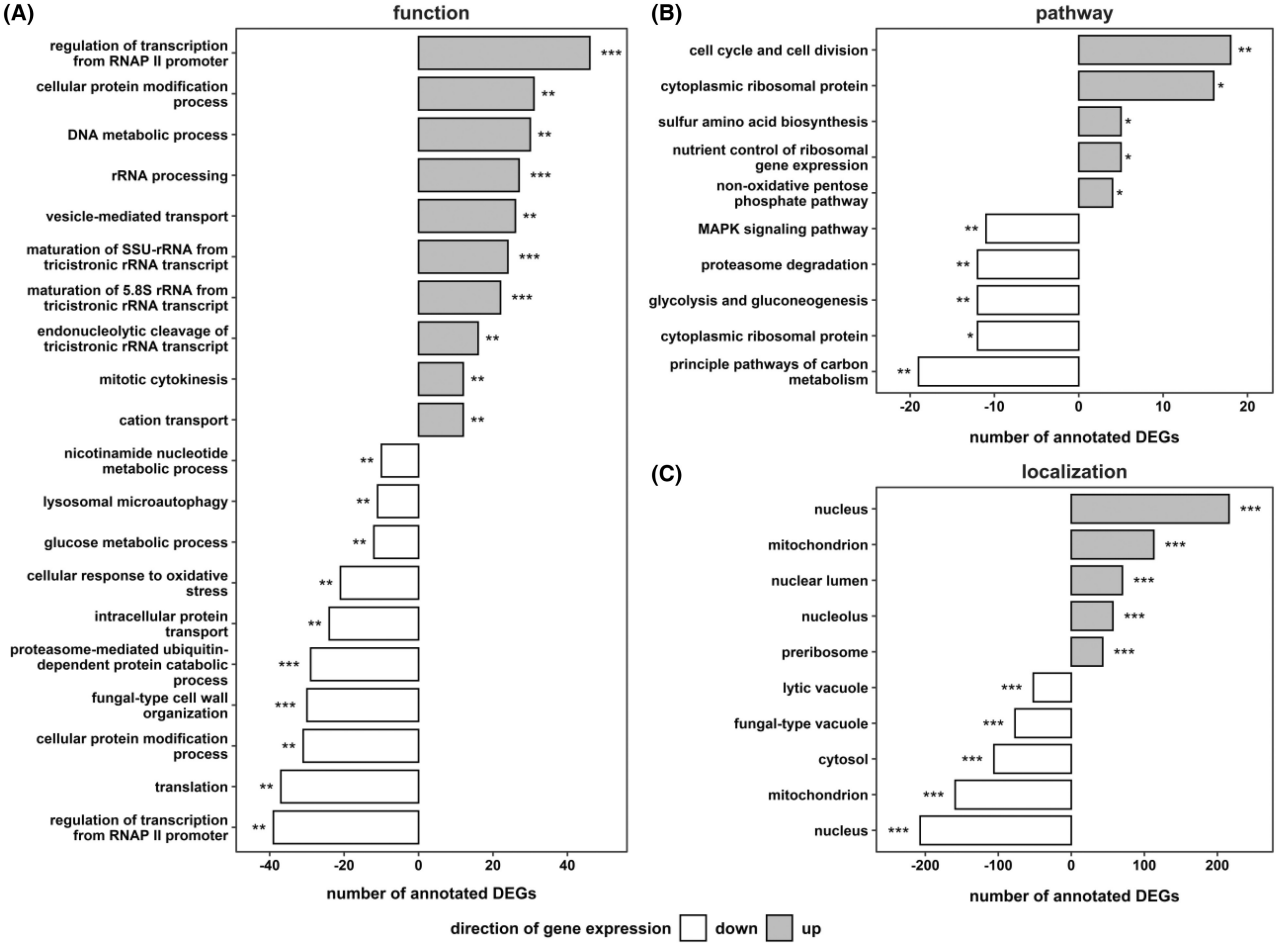
Functional enrichment analysis of steady‐state DS based on (A) biological function, (B) pathway affiliation and (C) cellular localization annotations. The false discovery rate (FDR) interval is indicated by asterisks for each GO term (* 1 × 10^−5^ ≤ FDR <5 × 10^−2^; ** 1 × 10^−10^ ≤ FDR <1 × 10^−5^; *** FDR < 1 × 10^−10^).

The transcriptomic landscape of yeasts adapted to unstable glucose uptake during DS was investigated by three‐level enrichment analysis. Gene ontologies grouping genes according to biological function, pathway affiliation, and compartment‐specific localization were used to characterize 728 repressed and 676 induced genes relative to RS (Figure 6). The dominant fraction of DEGs was operating in the nucleus, where highlighted reconstructions of the regulatory network and proliferation apparatus occurred. The first is apparent as 20% of both up‐ and down‐regulated mRNAs encoded transcription factors. More specifically, significant down‐regulation of nuclear protein quality control through ubiquitin‐dependent proteolytic activity and up‐regulation of cell cycle‐related DNA metabolic processes was observed.

Regarding the proliferative capabilities, the ‘cell cycle and cell division’ pathway were amplified by increasing expression levels of engaged cyclins, kinases and transcription factors. Attached were up‐regulated functional categories on the level of DNA repair and segregation and cell division, represented by the terms ‘DNA metabolic process’ and ‘mitotic cytokinesis’ respectively. Gene expression of the translational machinery was strongly induced at the stage of early ribosome biogenesis (RiBi) in the nucleus, including rRNA processing and the maturation of several ribosomal subunits (Woolford & Baserga, 2013). Induction of RiBi genes was accompanied by the up‐regulated ‘nutrient control of ribosomal gene expression’ ontology, which involved genes of the cAMP‐dependent protein kinase A (PKA) nutrient‐signalling network, such as the receptor protein Gpr1 and PKA subunits *TPK1/3*. On the other end of the ribosomal life cycle, down‐regulation of proteolytic activity was evident from several GO readouts, particularly represented by the term ‘proteasome‐mediated ubiquitin‐dependent protein catabolic process’. The ubiquitin system predominantly controls the nuclear turnover of ribosomal subunits and its activity must be repressed to allow atypical overexpression of RPs (An & Harper, 2020; Sung et al., 2016). Additionally, mature ribosomes were adjusted based on their subunit configuration in both the cytosol (16 up, 12 down) and the mitochondrion (7 up, 10 down).

Metabolic enzymes were primarily repressed in the regime‐transitioning environment of DS. Especially, glycolytic catabolism was subjected to a slowdown as represented by several enriched GO terms. One exception, however, was the non‐oxidative branch of the pentose phosphate pathway, possibly a reflection of increased anabolic needs to supply the overproducing translation machinery. Furthermore, *S. cerevisiae* sacrificed activity of various stress‐specific programs such as the mentioned MAPK signalling, the ‘cellular response to oxidative stress’ or the nutrient‐starvation‐specific ‘lysosomal microautophagy’ (Gross & Graef, 2020). In contrast, the up‐regulated biological function ontology ‘vesicle‐mediated transport’ involved many endocytic genes. Recently, Johnston et al. (2020) reported that under conditions of extracellular nutrient scarcity, yeasts scavenge for alternative nutrients via increased endocytosis activity.

**FIGURE 7:**
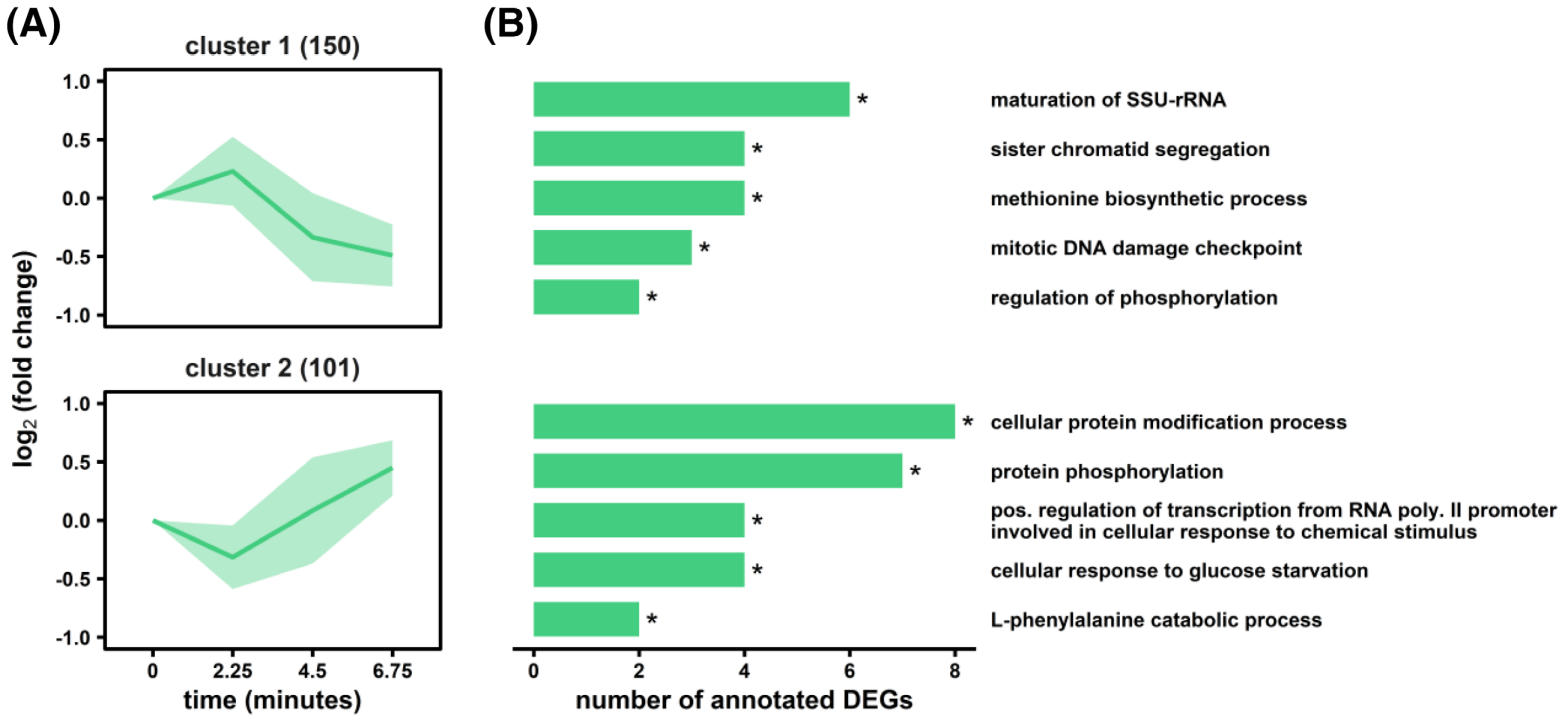
Differential gene expression analysis of the adapted r‐LSL time series. (A) Two clusters with similar gene‐expression dynamics are shown with the number of dedicated genes in brackets. (B) Corresponding gene ontology enrichment analysis. The false discovery rate (FDR) interval is indicated by asterisks for each GO term (* 1 × 10^−5^ ≤ FDR <5 × 10^−2^).

Complementary to the steady‐state assessment, we investigated the existence of persistent regulatory dynamics of the DS‐population. Accordingly, 251 stimulus‐responsive genes in fully adapted cells were identified (Figure 7). Two symmetric clusters revealed oscillatory gene expression changes with two inflection points during 9‐min r‐LSL cycles. With this short window of observation, the clusters were mainly enriched for fast‐responding genes with short half‐lives <10 min, such as those involved in stress response, ribosome biogenesis and transcription regulation (Miller et al., 2011). Especially, the latter two categories were also prevalent in the non‐adapted response, reflected by 142 overlapping genes accounting for 57% of the adapted DEG dynamic. Thus, despite pronounced changes in the global transcriptional landscape during steady‐state DS, *S. cerevisiae* still executes starvation‐induced short‐term gene expression changes that are independent of its adaptation status.

Cluster 1 revealed regulatory activity of the DNA replication process, represented by the GO terms ‘sister chromatid segregation’ and ‘mitotic DNA damage checkpoint’. The latter involved *RAD53*, the master effector kinase regulating progression through the S‐phase of the cell cycle (Branzei & Foiani, 2006). Recently, *RAD53* revealed additional transcriptional control over several promoters covering 20% of the whole yeast genome, emphasizing its wide regulatory influence (Sheu et al., 2021). Notably, there was no overlap with ‘cell cycle and cell division’ genes up‐regulated during steady‐state DS (Figure 6B) despite their involvement in the same signalling cascade of S‐phase DNA damage checkpoint, such as the mediator protein *RAD9* (Pardo et al., 2017). The ontology ‘methionine biosynthetic process’ confirms the existence of a tightly regulated crosstalk between glucose sensing and methionine synthesis. Zou et al. (2020) linked this relationship to the rate‐limiting function of methionine on translation initiation through the formation of methionyl tRNA. More differentially expressed kinase encoding genes were found in the two top GO terms in cluster 2, with no apparent functional connection to the unstable nutrient availability. In contrast, the following two entries contain regulatory proteins involved in the early starvation response (*USV1* and *MTL1*) or glucose catabolite repression, such as transcription factors Adr1 or Mig1 and the Snf1 subunit gene *SIP2* (Stasyk & Stasyk, 2019).

To recapitulate, repeated exposure to glucose shortage in LSL cycles induced pronounced transcriptional reprogramming in *S. cerevisiae*. The strategic response encompassed up‐regulated growth capacities and down‐regulated metabolic and stress‐responsive pathways. However, full adaptation did not shut down the repeated on–off switching of immediate tactical mechanisms involved in DNA replication and translation initiation control.

### A stress defence—growth trade‐off shapes the fate of yeasts in a heterogeneous environment

**FIGURE 8:**
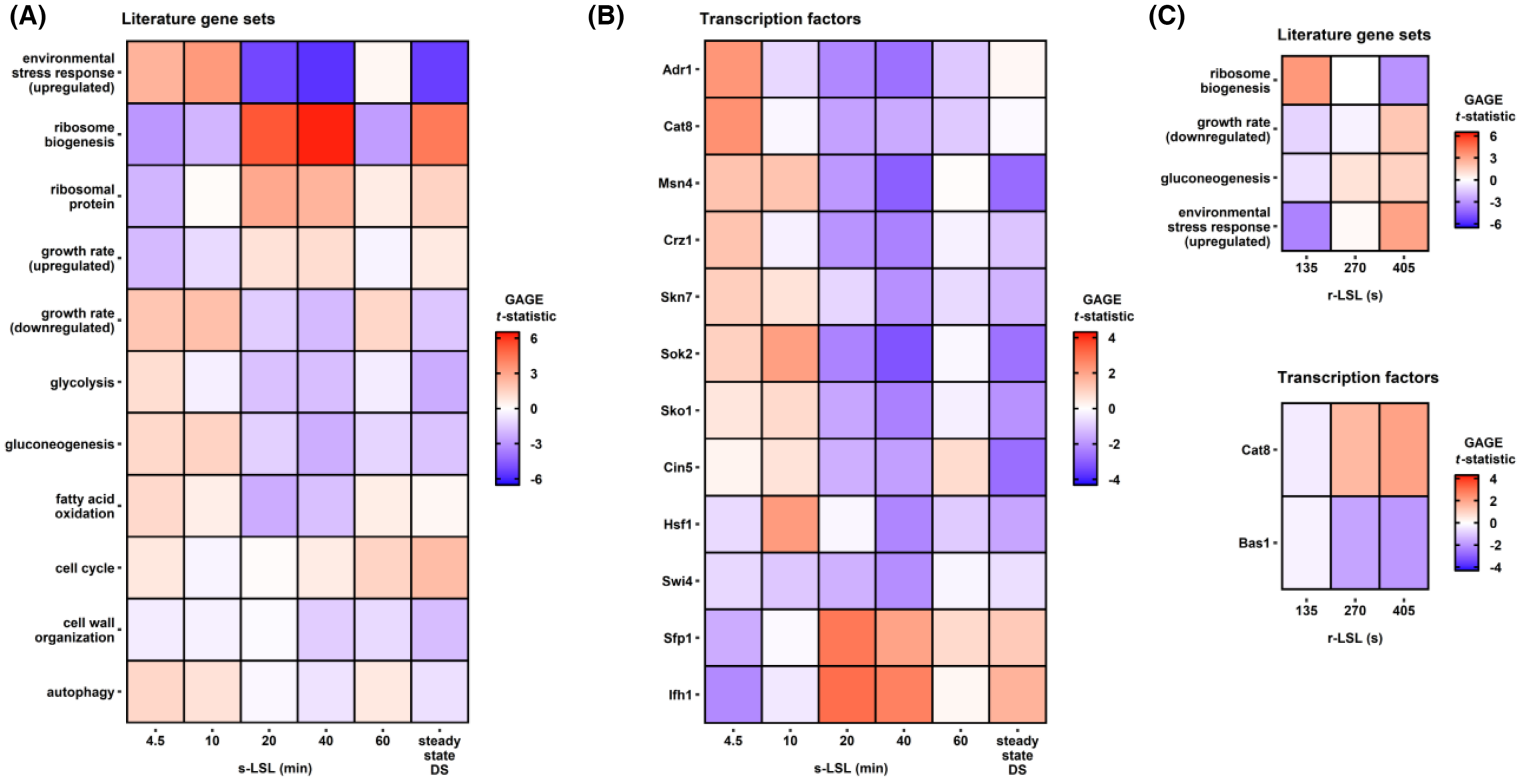
Gene set enrichment analysis (GSEA) of pre‐defined gene lists from literature and transcription factor target lists. The reported *t*‐statistic implies the strength and direction of coordinated differential gene expression of a given set. GSEA was performed comparing the single (s) LSL time series and steady‐state DS (A and B) on the one hand, and the dynamics within the repeated (r) LSL cycles (C) on the other hand. Only gene sets with significant enrichment during at least one sample point (FDR <0.05) are reported in this figure.

In the final part of this study, we investigated the presence of global transcriptional programs and their underlying regulatory mediation through gene set enrichment analysis (Figure 8). Non‐adapted yeast cells showed significant signs of executing the environmental stress response (ESR), a program that initiates a broad spectrum of stress‐responsive genes (ESR‐induced ESRi) while simultaneously repressing ribosomal protein (RP) and biogenesis (RiBi) genes (Brion et al., 2016; Gasch et al., 2017). This well‐investigated characteristic is clearly visible in Figure 8A and has been observed previously in various stresses (Levy et al., 2007; MacGilvray et al., 2020). The temporal dynamic of the ESR follows the earlier described trend of overshooting as evidenced by matching patterns of gene sets controlled by its master transcription factors Msn4, Sko1, Sok2 (ESRi), Sfp1 (RP and RiBi) and Ifh1 (RP) (Gasch et al., 2017; Gutin et al., 2015; Skoneczny, 2018). Besides common ESR regulators, we identified the activity of non‐ESR‐associated stress‐responsive TFs such as heat shock transcription factor Hsf1, the calcineurin‐responsive zinc finger Crz1, and the oxidative stress regulators Cin5 and Skn7. Interestingly, Hsf1 targets seem to operate ‘out of phase’ compared to the overall transcriptional dynamics suggesting divergent signal integration. Indeed, ESR coordination is dominated via target of rapamycin 1 (TORC1) and PKA crosstalk (López‐Maury et al., 2008), while glucose starvation‐induced Hsf1 phosphorylation is dependent on the Snf1 signalling cascade (Hahn & Thiele, 2004). We further assessed expression changes of 267 strictly growth rate‐dependent genes extracted from Fazio et al. (2008) which followed the observed transient $Y_{X/S}$ reduction implied by Figure 3A. In contrast, the cell cycle gene set was not affected significantly during the non‐adapted time series, even though Figure 8B indicated a steady gene expression decline of Swi4 targets. However, this cell cycle regulator reportedly plays a role in the induction of several stress‐responsive genes under the control of the Xbp1 promoter (Mai & Breeden, 1997). Altogether, we anticipate that stress‐sensing networks dominated the transfer of non‐adapted cells to a defensive state. We rule out mere growth rate sensing as an effector since μ correlated genes surged after 4.5 min, while the first significant reduction in $Y_{X/S}$ was observed 1 h post‐s‐LSL stimulus.

Remarkably, the adapted DS‐culture predominately followed the same course of transcriptional dynamics of the mid‐term s‐LSL response after 20–40 min. In this phase, the *S. cerevisiae* transcriptome ramped up growth‐associated genes and repressing stress‐responsive genes. Regarding metabolic gene sets a pronounced difference emerged: The non‐adapted response showed expression changes of gene sets representing glycolysis, gluconeogenesis and fatty acid oxidation coordinated by their respective TFs Adr1 and Cat8 (Young et al., 2003). In contrast, the DS‐phenotype showed down‐regulated glycolytic/gluconeogenic genes, but no sign of Adr1 or Cat8 regulation. Instead, Cat8 targets were constantly differentially expressed within adapted LSL‐cycling. Another regulatory program with persistent temporal activity during DS was controlled by Bas1, a control mechanism for ATP homeostasis (Pinson et al., 2019). Figure 8C further indicates additional short‐term dynamics of ESR‐associated gene expression, although to a lesser extent, compared to the time series after a single famine stimulus. Notably, only RiBi, not RP genes, were differentially expressed in concert with the ESRi group.

## DISCUSSION

### The impact of famine zones in industrial bioreactors

Gradients of limiting nutrients occur when reaction times of microbial activity match or exceed mean circulation times (Haringa et al., 2018; Lara et al., 2006). This correlation causes the appearance of carbon starvation regimes during the growth (Nadal‐Rey et al., 2021) or production phase of C‐limited fed‐batch processes. We imposed a single famine stimulus on steady‐state yeast cultures to investigate the influence of this scale‐up effect on strain performance when starvation zones start to build up. The population which was already adapted to glucose limitation apparently perceived the exposure to glucose starvation as a warning signal, which immediately triggered facets of the ESR (Gasch et al., 2000). Even though optimal conditions were restored within 76 s, *S. cerevisiae* CEN.PK 113‐7D obviously lacks the regulatory capability to stop the initiated program efficiently. Instead, the stressed cells shifted into a ‘panic mode’ which is characterized by frequent switching on/off of regulatory genes that caused increased ATP expenditure and impaired growth. Understanding the underlying regulatory mechanisms is paramount to engineer robust strains and guided this study.

Several studies anticipate that the initiation of the ESR following acute glucose starvation is dominated by cAMP‐dependent PKA signalling (De Wever et al., 2005; Görner et al., 2002; Martínez‐Pastor et al., 1996). PKA, in turn, controls the ESRi regulon through activation of the transcriptional inducers Msn2/Msn4 and inactivation of the repressors Sko1 and Sok2 (Gutin et al., 2015). A characteristic property of these and other stress‐related TFs such as Crz1 is their oscillating translocation between nucleus and cytoplasm (Zadrąg‐Tęcza et al., 2018). Gutin et al. (2019) reported that Msn2/Msn4 activate two successive bursts of transcription upon exposure to osmotic stress: First, PKA dephosphorylates Msn2/Msn4 causing their translocation to the nucleus to initiate quick but weak transcriptional changes within 10 min. Strong transcriptional changes require a pulsatile translocation of Msn2/Msn4 between nucleus and cytoplasm, during which nuclear export is mediated by Msn5. Thus, we reason that the non‐adapted response examined in this work displayed the initiation phase but not the second progression phase, potentially explaining the mild log‐fold changes compared to others (Causton et al., 2001; Gasch, 2007). Recently, Wu et al. (2021) inferred that Msn4, but not Msn2, is regulated by an incoherent feedforward loop (IFFL), including the intermediate regulator kinase Yak1. Since the purpose of IFFLs is to accelerate response time and execute oscillatory behaviour (Reeves, 2019) we interpret the absence of significant Msn2 regulation (Figure 7B) as further support for an early ESR retraction mechanism.

Recent research concerning the ESR identified strong counter‐correlated gene expression between the ESRi and RP/RiBi clusters. The latter, sometimes referred to as the ESRr (ESR repressed) cluster, is mediated by the regulatory activity of Sfp1, Ihf1/Fhl1 and the general activator/repressor TF Rap1 (Gasch et al., 2017; MacGilvray et al., 2020). Our experiment confirmed the mutual relationship between ESRi and ESRr, even though transcriptional control of RP and RiBi genes was executed exclusively via Sfp1 and Ifh1. Both TFs are inducers of proliferative capacity as Sfp1 binds the RiBi‐associated PAC promoter while Ifh1 positively controls RP gene expression through a currently unknown promoter architecture (Cipollina et al., 2008; Schawalder et al., 2004). Either TORC1 or PKA retains their active state during optimal growth. Sudden downshift of nutrients, however, induces PKA‐coordinated ESRr down‐regulation, which can be explained by cytosolic localization of Sfp1 and Ifh1 alone (Shore et al., 2021; Zencir et al., 2020). This exclusively stress‐specific role of Sfp1 and Ifh1 is mediated through their antagonizing TFs Dot6/Tod6 and Stb3 respectively (Huber et al., 2011; Plank, 2022).

Taken together, the observed retraction and overshooting gene expression originated from the TORC1/PKA circuitry since both nodes tune the temporal and local displacement of overlapping TFs. Acute glucose exhaustion signals PKA to execute its feedforward role to rapidly respond to the stimulus and override the steady‐state controller TORC1 (Kunkel et al., 2019). Similarly, PKA remains dominant when glucose levels elevate, leading to overshooting regulation until TORC1 regains control. It is somewhat surprising that the overshoot amplitude matches the initial response. Combined with the feedforward role of PKA, multiple feedback mechanisms exist with the potential to act as signal amplifiers. For instance, Ashe et al. (2000) reported severe inhibition of translation initiation within 30–60 s after glucose depletion, which can induce rapid RiBi and RP mRNA degradation (Huch & Nissan, 2014). In our experiment, ample nutrient conditions 2 min after the start of the s‐LSL cycle superimposed the initiated decay of translation‐associated genes. The phenotype may be explained by consequent disparate sensing of expected versus actual growth rates that may prompt yeasts to boost transcription of growth‐associated mRNAs causing the observed overshoot (Shore et al., 2021). Regarding the regulation of energy homeostasis, the Snf1 kinase is activated upon AEC drops by as narrow as 0.1 causing inhibition of TORC1 (González & Hall, 2017; Oakhill et al., 2012) and co‐phosphorylation of stress‐responsive PKA targets (De Wever et al., 2005).

Once activated, Snf1 co‐activated specific gene expression programs via crosstalk with the TOR/PKA node. Furthermore, the TFs Adr1 and Cat8 are amplified but not Mig1 (Busti et al., 2010). Besides Snf1, Mig1 is dependent on further activation through hexose kinase 2 and represents one branch of dual control over the carbon catabolite repression (CCR) regulon. The second branch integrates extracellular glucose signals through the sensory Rgt2/Snf3‐PKB system (Busti et al., 2010; Kim, Roy, et al., 2013). Since we did not observe any differentially expressed CCR genes, we reason that Snf1 regulation is solely AEC driven. Consequently, the strictly glucose‐related Rgt2/Snf3‐PKB pathway was not implicated in the non‐adapted response. Short‐term energy deprivation further induced changes in mitochondrial translation (see Figure 5 cluster 5). Yi et al. (2017) reported that Snf1 associates with the mitochondrial membrane to support respiratory activity for 10 h of glucose starvation—a prerequisite to sustain autophagy during arrested growth. We hypothesize the existence of a preparative program that was aborted in early stage in analogy to the observed ESR dynamics: Genes encoding translational capacities might have been differentially expressed as a preparatory measure to alter mitochondrial respiration. Nevertheless, the cascade was shut down promptly after return to steady‐state conditions.

### The transcriptional response mirroring frequent glucose starvation

Once famine zones are established during industrial fermentations, yeast cells require adaptation to withstand the repeated exposure to the starvation conditions that request regime transitions. Our experimental design enabled the investigation of the growth phenotype and the transcriptional strategy during oscillatory glucose availability by imposing an intermittent feeding regime. On a macroscopic level, the cellular mode of operation mimicked that of a faster‐growing population, *that is*, reduced carbon storage pools, increased rRNA content and ribosomal gene expression, decreased ESR expression levels, down‐regulated glycolytic genes and up‐regulated cell cycle genes (Brauer et al., 2008; Nissen et al., 1997; Regenberg et al., 2006; Silljé et al., 1999; Xia et al., 2022).

**FIGURE 9:**
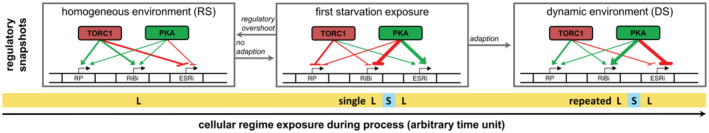
Key regulatory elements comprising target of rapamycin 1 (TORC1) and protein kinase A (PKA) signalling. DS, dynamic steady state; ESRi, induced environmental stress response genes; L, C‐limitation; RiBi, ribosome biogenesis genes; RP, ribosome protein genes; RS, reference steady state; S, C‐starvation.

Processing of dynamic environmental inputs can cause repeated decoupling of the growth rate from the expected μ‐specific transcriptome (Levy & Barkai, 2009; Zakrzewska et al., 2011; Zaman et al., 2009). Dedicated studies assigned this dissonance predominantly to high PKA activity, which is in agreement with our DS dataset: Strong ESRi repression and RiBi induction, backed by increased expression levels of PKA pathway components are opposed to relatively weak RP induction, owing to the TORC1‐dependency of the latter (see summarizing Figure 9) (Huber et al., 2011). Under the investigated conditions, however, cells did not shut down rapid translation initiation control mechanisms, which is also reflected by dynamic ESRi/RiBi patterns during the adapted time series. This finding may surprise as the yeast's ability to decelerate translation upon glucose scarcity may be regarded as a persistent ‘first line of defence’ (Hershey et al., 2012). Instead, cells apparently enable growth by benefitting from higher ribosome abundance as it was observed in other studies (Metzl‐Raz et al., 2017; Young & Bungay, 1973). This seems to be an evolutionarily conserved principle since bacterial cells elevate ribosome content for accelerating growth after relieving from various stresses (Bergen et al., 2021). However, despite amplifying genes encoding ribosomal proteins, yeasts further backed ribosomal biogenesis and configuration to maximize growth capacities. In this context, Parenteau et al. (2015) reported that perturbed growth can induce the expression of different subunits including gene paralogues which increase fitness and which are repressed under normal growth. Likely, de‐repressed RP paralogues do not exert stress‐specific functions but may enable atypical gene overexpression. In our study, however, we could not draw any conclusion if and to which extent differentially expressed paralogue genes actually contributed to the observed phenotype.

Furthermore, even though still under debate, increased RiBi expression supposedly indirectly promotes progression over START during the cell cycle through Whi5 inactivation (Bernstein et al., 2007; Polymenis & Aramayo, 2015; Schmoller et al., 2015). Eased START passaging leads to reduced time within the G0/G1 phase and decreased trehalose and glycogen pools (Brauer et al., 2008; Paalman et al., 2003). Hence, we argue that the cell cycle aligned with the PKA‐guided shaping of the translational machinery following the environmental signal as a feedback mechanism (Müller et al., 2003). Transcriptome analysis revealed added regulatory rearrangements that point towards a preference for PKA activity over TORC1 control. Down‐regulation of non‐relevant stress signalling cascades was observed, such as the osmo‐responsive MAPK cascade—a constitutive inhibitor of PKA (Mace et al., 2020). In terms of energy homeostasis, elevated translational capacity is ATP‐costly and might have contributed to the increased AEC difference during the LSL transition in DS. A more pronounced drop of the AEC, in turn, could potentially amplify the earlier discussed Snf1‐guided energy signal integration with positive feedback for PKA and repression of TORC1 targets. In conclusion, exposure to recurring regime transitions shifted the regulatory response of *S. cerevisiae* into a mode of dominating PKA signalling. The kinase constantly overrides the steady‐state controller TORC1 and is amplified by several feedback mechanisms, the consequence of which is a cellular tuning to enable efficient growth acceleration based on the adapted ribosome portfolio.

### Potential transfer of knowledge for industrial strain engineering

Understanding how cells adapt to substrate heterogeneities in industrial bioreactors is important for bioprocess optimization. The trade‐off between stress‐response and internal growth capacity turned out as a key mechanism to explain cellular performance under recurring glucose starvation. If biomass itself is the product, maintaining a high growth rate is a favourable trait. However, for exploiting metabolic production capacities the prioritization of re‐installing high growth rates may deteriorate the supply of carbon, reduction factors, and energy for the targeted product formation. This conflict may arise for metabolic products as well as for heterologous proteins. For the latter, ribosome buildup could potentially reduce the product yield and *vice versa* (Birnbaum & Bailey, 1991). Yet, predicting the impact of competing resource allocations influenced by environmental signalling is not a trivial task (Kafri et al., 2016). For instance, Wright et al. (2020) reported increased insulin production from *S. cerevisiae* in a two‐compartment scale‐down approach with a remarkable conformity to the results presented here: Environmental heterogeneity enforced the translational machinery and repressed stress‐responsive networks, which proved to be beneficiary for insulin productivity. In consequence, we propose two use cases for our dataset.

First, the deployed scale‐down approach can enable strain engineers to streamline industrial hosts. For instance, we observed a presumably unnecessary induction of the ESRi cluster upon first‐time glucose withdrawal as it was actively repressed during repeated glucose oscillations. Thus, deleting Msn2/4 could potentially save unwanted resource expenditure. This proposal is supported by the work of Ashe et al. (2000), who prove that *msn2/4Δ* strains abolished the induction of the stress response program while maintaining a normal growth phenotype. Likewise, our dataset suggests wasteful gene expression induced via Hsf1 and Crz1. Indeed, altering nuances of the regulatory response via TF engineering gains popularity as relatively minor changes in the genetic background can improve strain performance significantly (Mohedano et al., 2022). For instance, several studies achieved increased ethanol yield through the atypical expression of just a single transcription factor (Michael et al., 2016; Samakkarn et al., 2021; Watanabe et al., 2017).

Second, this and other work supports the finding that glucose availability, but also other industrially relevant heterogeneities, converge mainly on the level of PKA signaling (de Lucena et al., 2015; De Melo et al., 2010; Norbeck & Blomberg, 2000; Zaman et al., 2009; Zhao et al., 2015). To conclude, we would like to formulate a somewhat alternative, maybe even provocative scale‐down route. If mere activation/inhibition dynamism of PKA shapes the corpus of adaptation effects during industrial fermentations, wouldn't triggering PKA according to process‐relevant stimuli suffice as the most simplistic scale‐down experiment? Instead of trying to mimic physicochemical perturbations by wet‐lab approaches as close to reality as possible, it might be sufficient to characterize the frequency and amplitude of relevant stimuli a priori, for instance, by means of CFD simulations. Consequentially, the simulation output should be translated into an input signal for the PKA hub. Tools to control PKA activity on relevant scales are already available, such as optogenetic switches (Hepp et al., 2020; Stewart‐Ornstein et al., 2017). Ultimately, this approach could empower rational scale‐down by providing a fast and easy method to estimate the impact of extracellular signal fluctuations on strain performance.

## CONCLUSIONS

This study revealed that perception of extracellular glucose concentration alone can induce pronounced biological scale‐up effects. Industrially relevant glucose gradients with regime transitions between carbon limitation and starvation were set in a chemostat with intermittent feeding. The single most prominent observation, irrespective of the adaptation status, was the adjustment of internal resources following a growth–stress response tradeoff. Interpretation of transcriptomic data allowed us to identify the implication of several regulatory circuits, all centred around protein kinase A. In consequence, we were able to define engineering propositions with the potential to (i) improve strain performance in an industrial setting and (ii) simplify classical scale‐down. Here, a growth scenario was investigated with the laboratory *S. cerevisiae* strain CEN.PK113‐7D. Comparative experiments carried out under the same premise with industrial production hosts, especially considering polyploid strains, could shed further light on the general applicability of the demonstrated approach.

## AUTHOR CONTRIBUTIONS

**Steven Minden:** Conceptualization (lead); data curation (lead); formal analysis (lead); investigation (lead); methodology (lead); writing – original draft (lead); writing – review and editing (equal). **Maria Aniolek:** Formal analysis (supporting). **Henk Noorman:** Project administration (equal); writing – review and editing (equal). **Ralf Takors:** Funding acquisition (lead); project administration (equal); resources (lead); supervision (lead); writing – review and editing (equal).

## FUNDING INFORMATION

This research was supported by the German Federal Ministry of Education and Research (BMBF), grant number: FKZ 031B0629. S.M. is supported by ERA CoBioTech/EU H2020 project (grant 722361) ‘ComRaDes’, a public–private partnership between the University of Stuttgart, TU Delft, University of Liege, DSM, Centrient Pharmaceuticals and Syngulon.

## CONFLICT OF INTEREST

The authors declare no conflict of interest.

## Supporting information


Appendix S1

Appendix S2

## Data Availability

The data that support the findings of this study are available from https://dataverse.nl/dataverse/minden‐microbialbiotechnology.
