# Supplementary material for: Performing in spite of starvation: How Saccharomyces cerevisiae maintains robust growth when facing famine zones in industrial bioreactors
Source: Microb Biotechnol. 2022 Dec 8;16(1):148–68. doi: 10.1111/1751-7915.14188 (PMC9803336; doi:10.1111/1751-7915.14188)
Supplement: Supplementary file 1 — Appendix S1 [file MBT2-16-148-s002.docx]

**Performing in spite of starvation: How *Saccharomyces cerevisiae* maintains robust growth when facing famine zones in industrial bioreactors**

Steven Minden^1^ | Maria Aniolek^1^ | Henk Noorman^2,3^ | Ralf Takors^1,*^

^1^Institute of Biochemical Engineering, University of Stuttgart, Allmandring 31, 70569 Stuttgart, Germany

^2^Royal DSM, 2613 AX Delft, The Netherlands

^3^Department of Biotechnology, Delft University of Technology, 2628 CD Delft, The Netherlands

**Correspondence:** Institute of Biochemical Engineering, University of Stuttgart, Allmandring 31, 70569 Stuttgart, Germany. Tel: +49 711 685 64535; Fax: +49 711 685 55164; E-mail: [takors@ibvt.uni-stuttgart.de](mailto:takors@ibvt.uni-stuttgart.de)

**Funding information:** German Federal Ministry of Education and Research, grant number: FKZ 031B0629; ERA CoBioTech/EU H2020 project grant number: 722361 (ComRaDes)

**A1 BIOREACTOR CHARACTERIZATION**

The ungassed power number $N_{p}$ was determined based on a Reynolds number of 6 · 10^4^. Next, the ungassed power input was calculated under the assumption that both Rushton turbines did not influence each other, yielding a value of 18.8 kg ⋅ m^2^ ⋅ s^−3^. Michel and Millers correlation was used to estimate the gassed power input of 12.0 kg ⋅ m^2^ ⋅ s^−3^, translating to a volumetric gassed power input of 7.1 W ⋅ kg^−1^ (Michel et al., 1962).

Estimation of the circulation time $t_{c}$ was based on the approach reported by Jüsten et al. (1996) with an estimated gassed power number $N_{p,g}$ of 48 according to the relation from (EKATO Rühr-und Mischtechnik GmbH 2013, page 52). The approach yielded time $t_{c}=0.1$ s allowing to conclude a perfectly mixed environment.

**Table S1:** Bioreactor operating figures and physical conditions.

| **parameter** | **symbol** | **value** | **dimension** | **reference** |
| --- | --- | --- | --- | --- |
| stirrer speed | $N$ | 13.3 | s^−1^ | set point |
| vessel diameter | $D_{v}$ | 0.125 | m | measurement |
| impeller blade width | $W$ | 1.5 · 10^−2^ | m | measurement |
| impeller diameter | $D_{i}$ | 6 · 10^−2^ | m | measurement |
| number of blades | $z$ | 6 | - | measurement |
| Number of impellers | $i$ | 2 | - | set point |
| liquid volume | $V_{l}$ | 1.7 · 10^−3^ | m^3^ | set point |
| volumetric air flow rate | $Q_{air}$ | 1.42 · 10^-5^ | m^3^ · s^−1^ | set point |
| broth density | $\rho$ | 1021 | kg · m^−3^ | measurement |
| dynamic broth viscosity | $\eta$ | 7.97 · 10^−4^ | kg · m^−1^· s^−1^ | assumed as H_2_O (30 °C) |
| ungassed power number | $N_{p}$ | 5 | - | (Bates et al., 1963) |
| constant for ${Po}_{g}$ | $c$ | 0.812 | - | (Taghavi, 2010) |

**Table S2:** Calculated parameters and formula.

| parameter | symbol | formula | dimension | reference |
| --- | --- | --- | --- | --- |
| Reynolds number | $Re$ | $N\cdot{D_{i}}^{2}\cdot\frac{\rho}{\eta}$ | - | (Bates, 1963) |
| ungassed power input | $Po$ | $i\cdot N_{P}\cdot{\rho\cdot D_{i}}^{5}\cdot N^{3}$ | kg ⋅ m^2^ ⋅ s^−3^ | (EKATO Rühr-und Mischtechnik GmbH, 2013) |
| gassed power input | ${Po}_{g}$ | $c\cdot\left( \frac{{Po}^{2}\cdot N\cdot D_{i}^{3}}{Q_{air}^{0.56}} \right)^{0.43}$ | kg ⋅ m^2^ ⋅ s^−3^ | (Michel, 1962) |
| Froude number | $Fr$ | $N^{2}\cdot\frac{D_{i}}{9.78}$ | - | (EKATO Rühr-und Mischtechnik GmbH, 2013) |
| aeration number | $N_{ae}$ | $\frac{Q_{air}}{N\cdot D_{i}^{3}}$ | - | (EKATO Rühr-und Mischtechnik GmbH, 2013) |
| gassed power number | $N_{p,g}$ | $z\cdot\frac{\left[ N_{p}+\left( 187\cdot N_{ae}\cdot{Fr}^{-0.32}\cdot\left( \frac{D_{i}}{D_{v}} \right)^{1.53} \right)-\left( 4.6\cdot{N_{ae}}^{1.25} \right) \right]}{\left[ 1+\left( 136\cdot N_{ae}\cdot\left( \frac{D_{i}}{D_{v}} \right)^{1.14} \right) \right]}$ | - | (EKATO Rühr-und Mischtechnik GmbH, 2013) |
| ungassed flow number | $N_{Fl}$ | $\left( 0.91\cdot N_{p}\cdot\frac{W}{D_{i}} \right)^{0.5}$ | - | (Jüsten, 1996) |
| gassed flow number | $N_{Fl,g}$ | $N_{Fl}\cdot\left( \frac{N_{p,g}}{N_{p}} \right)$ | - | (Jüsten et al., 1998) |
| circulation time | $t_{c}$ | $\frac{V_{l}}{N_{Fl,g}\cdot N\cdot D_{i}^{3}}$ | s | (Jüsten, 1998) |

**A2 CARBON, NITROGEN AND ELECTRON BALANCING**


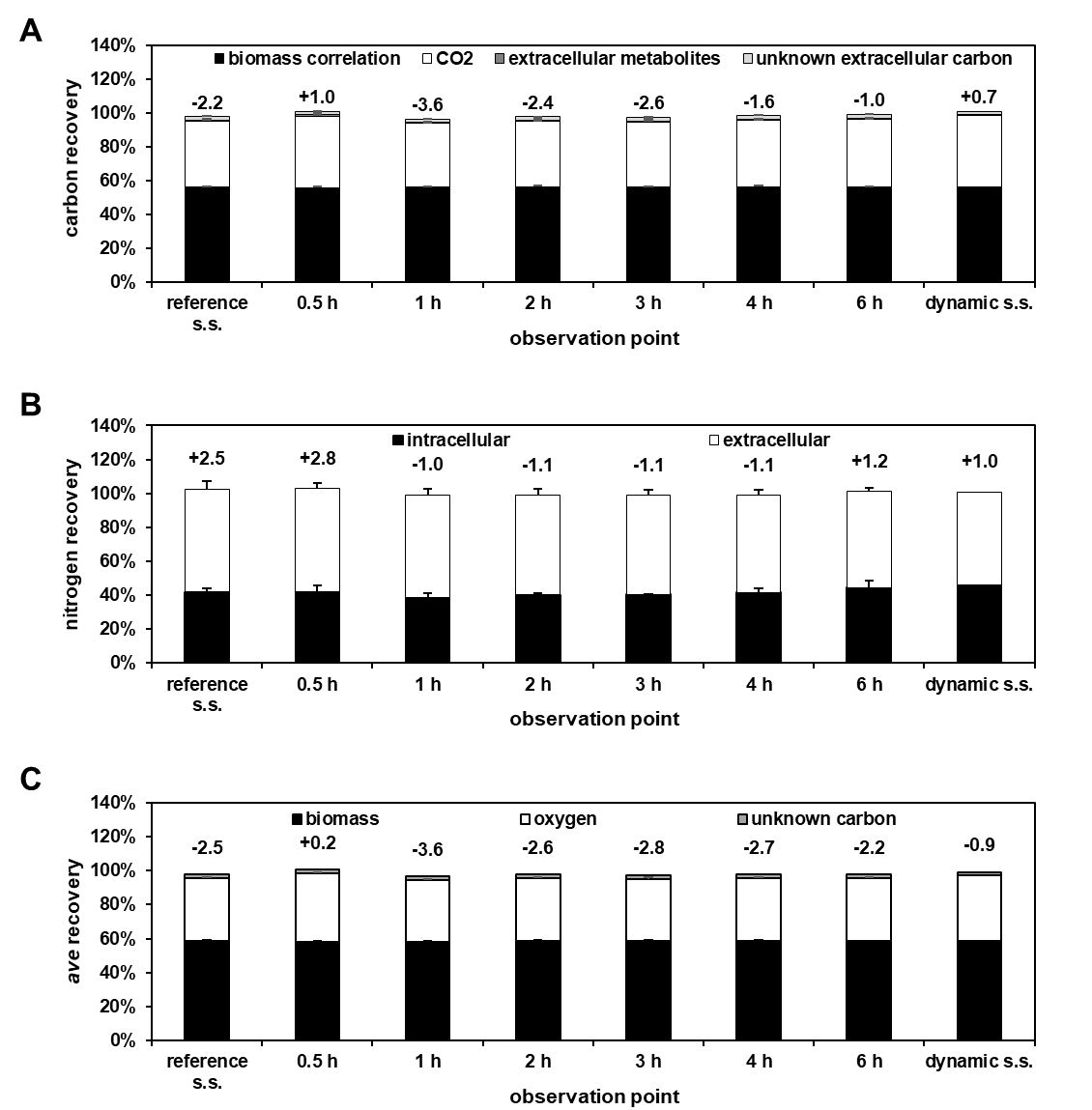


**Figure S1.** Recoveries of organic carbon (A), nitrogen (B) and available electrons (C) during the reference steady state up to 6 h post stimulus during the non-adapted time series and during the dynamic steady state. Numbers above bars indicate the deviation from complete recovery in percent. *ave*, available electrons.

**A3 DIFFERENTIAL GENE EXPRESSION ANALYSIS**

For gene expression analysis, the complete count table was split into three datasets: (i) the post s-LSL time series including the reference steady state (RS) and all time points of the series up to 3 h (figure S2), (ii) the adapted time series with timely equidistant samples within one r-LSL cycle of 9 min with time point 0 s serving as the reference sample (figure S3) and (iii) the characterization of the dynamic steady state (DS) using the grouped samples of RS and relaxed time points 2 h and 3 h as the reference and all samples of the adapted time series representing the adapted steady state. All functions used in the subsequent section were called from the R package DESeq2 v. 1.32.0 (Love et al., 2014). Cook’s distances were computed for an initial outlier detection via calling "cooks" from the “DESeqDataSetFromMatrix” object of the respective dataset. In order to discriminate between the introduction of biological and technical variance, we included the variables time (for biological variance), replicate (technical) and libseq (technical, merged variable for different library preparation and sequencing runs, see supporting information B; tab 1) into the model. Next, count tables were transformed into the rlog space to stabilize the variance of genes with low counts using “rlog”. Principal component analysis (plotPCA) revealed a strong influence of technical variables (figures S2 B+D; S3 B+D and S4 A+C). Thus, we applied a batch correction using “removeBatchEffect” from the limma v. 3.48.3 package (Ritchie et al., 2015) to dampen the technically introduced variance leading to a reduced model, which allowed the investigation of biologically introduced variance (figures S2 C+E; S3 C+E and S4 B+D).





**Figure S2**. Analysis of all 24 samples from the non-adapted post s-LSL time series. (A) Boxplot of the Cook’s distances. Numbers behind the sample time points indicate the biological replicate (B) Individual samples of 8 time points plotted on principal component 1 (PC1) and 2 (PC2). Labels R1 ‑ R5 indicate separate library preparation and/or sequencing runs. (C) Analogue to (B), but with the reduced model. (D) Corresponding scree plot of (B). (E) Corresponding scree plot of (C).





**Figure S3** Analysis of all 12 samples from the adapted r-LSL time series. (A) Boxplot of the Cook’s distances. Numbers behind the sample time points indicate the biological replicate (B) Individual samples of 4 time points plotted on principal component 1 (PC1) and 2 (PC2). Labels R1 - R5 indicate separate library preparation and/or sequencing runs. (C) Analogue to (B), but with the reduced model. (D) Corresponding scree plot of (B). (E) Corresponding scree plot of (C).





**Figure S4.** Analysis of all 36 samples generated in this study. (A) Individual samples plotted on principal component 1 (PC1) and 2 (PC2). Labels R1 ‑ R5 indicate separate library preparation and/or sequencing runs. (B) Analogue to (B), but with the reduced model. (C) Corresponding scree plot of (A). (D) Corresponding scree plot of (B).

**A4 Temporal dynamics of amino acids during the non-adapted response**

Intracellular amino acids were monitored via LC-MS/MS and steady state concentrations in RS versus DS have been published previously (Minden et al., 2022). The time series data for S-LSL was not published in the mentioned work due to the generally large variance in the dataset. However, in order to support the findings of figure 5 (dynamics of cluster 2) time series data of all measured amino acids is reproduced here. Amino acids where respective biosynthetic genes were found to be significantly enriched in cluster 2 (supporting information B, tab 5) are phenylalanine, glutamine, aspartic acid, methionine, leucine, histidine, tryptophan and arginine, all of which were quantified except histidine.





**Figure S5.** Dynamics of intracellular amino acids for three hours following a single transition into a starvation scenario (“feed off” phase) representing the non-adapted response (post s-LSL). The trends are shown as means (line) ± standard deviation (light area) of three biological replicates. The underlying single replicates are shown as grey symbols, where each symbol (square, triangle, circle) represents one biological replicate.

**REFERENCES**

Bates, R. L., Fondy, P. L., & Corpstein, R. R. (1963). An examination of some geometric parameters of impeller power. *Industrial and Engineering Chemistry Process Design and Development*, *2*(4), 310–314. https://doi.org/10.1021/i260008a011

EKATO Rühr-und Mischtechnik GmbH. (2013). *EKATO. THE BOOK*. (W. Himmelsbach & M. Delbrück, Eds.). Weinheim: Wiley-VCH Verlag GmbH & Co. KGaA.

Jüsten, P., Paul, G. C., Nienow, A. W., & Thomas, C. R. (1996). Dependence of mycelial morphology on impeller type and agitation intensity. *Biotechnology and Bioengineering*, *52*(6), 672–684. https://doi.org/10.1002/(SICI)1097-0290(19961220)52:6<672::AID-BIT5>3.0.CO;2-L

Jüsten, P., Paul, G. C., Nienow, A. W., & Thomas, C. R. (1998). Dependence of Penicillium chrysogenum Growth, Morphology, Vacuolation, and Productivity in Fed-Batch Fermentations on Impeller Type and Agitation Intensity. *Biotechnology and Bioengineering*, *59*(6), 762–775. https://doi.org/https://doi.org/10.1002/(SICI)1097-0290(19980920)59:6%3C762::AID-BIT13%3E3.0.CO;2-7

Love, M. I., Huber, W., & Anders, S. (2014). Moderated estimation of fold change and dispersion for RNA-seq data with DESeq2. *Genome Biology*, *15*(12), 1–21. https://doi.org/10.1186/s13059-014-0550-8

Michel, B. J., & Miller, S. A. (1962). Power requirements of gas‐liquid agitated systems. *AIChE Journal*, *8*(2), 262–266. https://doi.org/10.1002/aic.690080226

Minden, S., Aniolek, M., Sarkizi, C., Hajian, S., Teleki, A., Zerrer, T., … Takors, R. (2022). Monitoring Intracellular Metabolite Dynamics in Saccharomyces cerevisiae during Industrially Relevant Famine Stimuli. *Metabolites*, *12*(263), 1–26. https://doi.org/https://doi.org/10.3390/metabo12030263

Ritchie, M. E., Phipson, B., Wu, D., Hu, Y., Law, C. W., Shi, W., & Smyth, G. K. (2015). Limma powers differential expression analyses for RNA-sequencing and microarray studies. *Nucleic Acids Research*, *43*(7), e47. https://doi.org/10.1093/nar/gkv007

Taghavi, M. (2010). Power Consumption and Flow Regime Transition in a Stirred Tank Reactor, 25–28.
